# Neglected Tropical Diseases among Two Indigenous Subtribes in Peninsular Malaysia: Highlighting Differences and Co-Infection of Helminthiasis and Sarcocystosis

**DOI:** 10.1371/journal.pone.0107980

**Published:** 2014-09-23

**Authors:** Soo Ching Lee, Romano Ngui, Tiong Kai Tan, Roslan Muhammad Aidil, Yvonne Ai Lian Lim

**Affiliations:** Department of Parasitology, Faculty of Medicine, University of Malaya, Kuala Lumpur, Malaysia; The George Washington University Medical Center, United States of America

## Abstract

Soil-transmitted helminth (STH) infections have been documented among these minority groups since 1938. However the prevalence of STH is still high among these communities. Most studies tend to consider the Orang Asli (indigenous) as a homogenous group. In contrary, different subtribes have their own cultural practices. To understand this variation better, we studied the prevalence and associated factors of STH and other gut parasitic infections among two common subtribes (i.e. Temuan and Temiar). Results showed that the prevalence of the overall STH infections was higher in the Temuan subtribe (53.2% of 171) compared to the Temiar subtribe (52.7% of 98). *Trichuris trichiura* (46.2%) was the most prevalent parasite in the Temuan subtribe, followed by *Ascaris* spp. (25.7%) and hookworm (4.1%). In contrast, *Ascaris* spp. (39.8%) was more prevalent among the Temiar subtribe, preceded by *T. trichiura* (35.7%) and finally hookworm (8.3%). There were also co-infections of helminthiasis and intestinal protozoa among both Temuan and Temiar subtribes with rates being three times higher among the Temiar compared to Temuan. The most common co-infection was with *Entamoeba histolytica/dispar/moshkovskii* (n = 24; 24.5%, 16.0–33.0), followed by *Giardia* spp. (n = 3; 3.1%, −0.3–6.5). In Temuan, STH infection individuals were also infected with *Entamoeba histolytica/dispar/moshkovskii* (n = 11; 6.4%, 5.0–13.8), *Cryptosporidium* spp. (n = 3, 1.8%, −0.2–3.8) and *Giardia* spp. (n = 2, 1.2%, −0.4–2.8). In comparison, there was no *Cryptosporidium* spp. detected among the Temiar. However, it was interesting to note that there was an occurrence of co-infection of intestinal helminthiasis and sarcocystosis (intestinal) in a Temiar individual. The last report of sarcocystosis (muscular) among the Orang Asli was in 1978. The present study highlighted the importance of understanding the variation of infections amongst the different Orang Asli subtribes. It is vital to note these differences and use this knowledge to customise effective control measures for the various subtribes.

## Introduction

Soil-transmitted helminth (STH) infections are among the neglected tropical diseases that are highly prevalent among the indigenous (Orang Asli) of peninsular Malaysia [1], [2]. The Orang Asli constitutes approximately 0.6% of the national population and comprises 18 subtribes which are broadly classified under three major ethnolinguistic categories (i.e. Negrito, Senoi and Aboriginal Malay) [3]. The largest group of Orang Asli is Senoi, followed by Aboriginal Malay and Negrito. Generally, Senoi tribe is further divided into Temiar, Semai, Mah Meri, Che Wong, Ja Hut and Semoq Beri, whilst Aboriginal Malay consists of Temuan, Semelai, Temok, Jakun, Orang Kanaq and Orang Seletar and finally Negrito includes Kintaq, Lanok, Kensiu, Jahai, Mendriq and Beteq. Most of these indigenous communities work as swidden cultivators, hunters, collectors of forest resource, fishermen and wage labours [4]. Although the earliest record of STH infections (i.e., *T. trichiura*, *Ascaris* spp., hookworm) among the Orang Asli was more than 75 years ago [5], it is appalling to note that some of the recent studies have shown that these infections have failed to decline significantly with some indicating a prevalence rate of 100% [6].

Another important health concern among the Orang Asli is the high occurrence of multiparasitism in these communities. Co-infections of soil-transmitted helminth with a diversity of protozoa such as *Giardia* sp., *Entamoeba* spp., *Blastocystis* spp. and microsporidia with prevalence ranging from 15.0 to 22.2% have been observed [7]–[9]. Multiparasitism has proven to increase anaemia, morbidity and reduce cognitive development in children [10]–[12].

Aside from the common intestinal parasites mentioned above, there are some minor intestinal parasites that have been detected sporadically among the Orang Asli communities. These included *Chilomastix mesnili* [13], [14], *Iodamoeba butschlii* [13]–[15] and *Sarcocystis* spp. [16], [17].

Although there are many studies among the Orang Asli, interestingly not many have emphasized on the diversity of the Orang Asli subtribes. Most studies have regarded them as a homogenous group by not indicating the subtribe being studied or they have focused on only one subtribe. As there are 18 different subtribes and these subtribes have differing culture, attitude, behaviour and influenced knowledge which may affect the proper hygienic practices and their perception of diseases [18], it is therefore important to consider these variation especially when administering treatment intervention or anti-helminthic strategies.

Thus far, there were only four available reports that took into consideration the variation of parasitic infections in different subtribes [e.g. intestinal parasitic infections [13], giardiasis [19], ameobiasis [20] and blastocystosis [21]]. There is scarcity of information on STH and their co-infections with protozoan parasites in accordance to subtribes. Since STH infections are still prevalent, studies should be carried out to determine whether there is a variation of infection rates or co-infection with other protozoan based on the different subtribes in order to customise treatment strategies. This will facilitate a more effective strategy of delivering treatment where it is most needed. Hence, the objective of this current study was to determine the prevalence of STH and their co-infections with protozoan parasites among the two most common indigenous subtribes (i.e. Temuan and Temiar) of peninsular Malaysia.

## Materials and Methods

### Ethical consideration

The present study has been approved and granted the ethical considerations (i.e., MEC Ref. No. 824.11) by the Ethics Committee of the University Malaya Medical Centre (UMMC) Malaysia prior to the commencement of the study. Besides, the field work was approved by the Department of Orang Asli Development (JAKOA) and permission was obtained from the Tok Batin (chieftain) before entering the villages. During the briefing, the villagers were informed that their personal information was kept confidential and they have the right to withdraw from the study anytime without indicating any reasons. Their consent was obtained in written form either in signature or thumbprint. Parents signed on behalf of their children who were less than 12 years old.

### Study area

**Figure 1:**
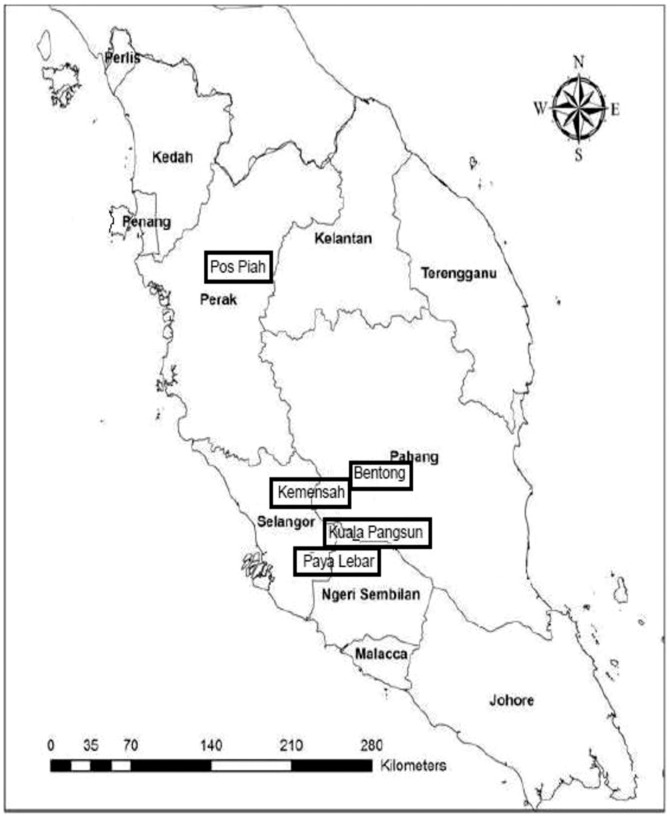
Location of the sampling sites.

A cross sectional study based on two different subtribes was conducted from December 2010 to April 2012 among five different villages in Peninsular Malaysia [i.e. Kuala Pangsun (101.88°E longitude, 3.21°N latitude), Bentong (101.92°E longitude, 3.53°N latitude), Kemensah (101.78°E longitude, 3.23°N latitude), Pos Piah (101.07°E longitude, 4.92°N latitude), Paya Lebar (101.9°E longitude, 3.14°N latitude)]. The locations of these villages were shown in Figure 1. These states experience humid tropical climate throughout the whole year.

### Sample collection and questionnaire

Before stool sample collection, a short oral briefing was given to explain the objective of this study. After the explanation, screw-capped containers labelled with names were distributed to the participants. Parents were advised to check that their children put samples into the correct containers. Containers with participants' samples were assembled back the following day. The participants were requested to answer questionnaire that include demographic data (i.e., age, gender and education), socioeconomic (i.e., occupation and household income), behavioural (i.e., personal hygiene such as defecation places and practice of taking bath in the river), environmental sanitation and living condition characteristics (i.e., type of water supply, drink boiled/unboiled water, latrine and garbage disposal systems). For those children who were less than 12 years old, parents or guardians answered on their behalf.

### Stool examination

Stools samples collected in the screw-capped containers were preserved in 2.5% potassium dichromate and stored at 4°C. For parasitological examination, formalin-ether concentration technique was used. Briefly, approximately 1 g to 2 g of stool samples were mixed with 7 ml of 10% formalin and 3 ml ethyl acetate in 15 ml falcon tubes. The samples were then centrifuged at 2500 rpm around 5 mins. Fecal smears were made from the sediment, stained with 0.85% iodine and observed by light microscope under the magnification of 100X and 400X for intestinal parasites. In addition, for the detection of *Cryptosporidium* spp., fecal samples were examined with acid-fast stain, (ie., Ziehl Neelsen stain) under the magnification of 1000X. All results were then recorded for data analysis. Hardcopies of the intestinal parasitic infection results were then sent to the Department of Orang Asli Development (JAKOA) and the Indigenous Health Unit, Ministry of Health, Malaysia. Specific treatment or effective control program were administered by these respective agencies.

### Statistical analysis

Data was analysed using the SPSS software (Statistical Package for the Social Science) programme for windows version 21 (SPSS Inc., Chicago, IL, USA). For descriptive analysis, percentage was used to define the general characteristics of the indigenous communities, prevalence of soil-transmitted helminth (STH) infections and intestinal protozoa. A Pearson's Chi-square test was used to check the associations between the dependent variables (i.e., prevalence of the STH infections) and independent variables (i.e., demographic data, socioeconomic, behavioural, environmental sanitation and living conditions) in this study. Any independent variables that showed *p* value less than 0.25 in the univariate model were included in the logistic regression analysis using forward likelihood ratio (LR) to identify the risk factors for STH infections. The risk factors were tested using Odds-Ratio (OR) and the significance was analyzed using 95% confidence interval and *p* value. The level of significance was defined as *p*<0.05.

## Results

### Temuan and Temiar subtribes: Socio-demographic

In the present study, two of the Orang Asli subtribes, namely Temuan and Temiar were included. Temuan subtribe converse in languages closely related to Malay and they are classified under the Aboriginal Malay tribe [4]. The distribution of Temuan is mainly at the lowlands of peninsular Malaysia close to the urban areas, such as states of Malacca, Negeri Sembilan, Pahang, Selangor and Johor. In the present study, Temuan participants were from the states of Selangor (ie., Kuala Pangsun and Paya Lebar, Kemensah) and Pahang (ie., Bentong).

As for the Temiar subtribe, they speak the Austro-asiatic language and is categorized under the Senoic tribe [13]. Generally, Temiar live close to or within forested areas, from central of Perak to northeastern part of the state. Unlike Temuan, the Temiar in the current study live in the northern part of the interior highlands of upper Perak state (ie., Pos Piah). This hilly village is located approximately 60 km from the town area of Sungai Siput, Perak. The people depended on the generator for electricity supply. Due to lack of transportation accessibility, the Temiar seldom travel out of their village and only a minority of them work in town.

A total of 269 samples were collected comprising of 171 from Temuan subtribe and 98 from Temiar subtribe. Due to logistic and financial constraints to access the interior communities, Temuan sample size was larger than Temiar in the current study. Of the 269 human samples, only 255 samples had demographic data, with 14 missing data. Overall, participants ranged from 1 to 70 years old with a median age of 14 years (IQR = 8–33.5). For Temuan subtribe, the participants ranged from 5 months to 70 years old (median age: 16.5 years old, IQR: 7–33); for Temiar subtribe, the range is from 1 to 60 years old (median age: 11 years old, IQR: 9–34).

Among the Temuan working adults, 37.5% (out of 48) were rubber tappers, 33.3% were farmers, 16.7% were factory workers and the rest were government workers, self-employed businessmen and drivers (4.2% each). Out of the 19 working Temiar adults, 78.9% were working as farmers and 21.2% were working as government servants.

Generally, majority of the participants in Temuan and Temiar subtribes were male (Temuan, 52.5% of 162; Temiar, 50.5% of 93), having formal education (59.9%; 64.5%), low household income which was less than RM500 (57.4%; 64.5%), unemployed (69.1%; 79.6%), boil water before consumption (80.2%; 98.9%), provided with latrine 69.8%; 94.6%), defecate indiscriminately (51.9%; 89.2%), take bath in the river (36.4%; 83.9), dispose garbage at a proper place (61.6%; 86.0%), own pets (63.6%; 86.0%) and wear shoes outside (69.8%; 100.0%). Detailed sociodemographic characteristics of the study population based on different subtribes were shown in Table 1.

**Table 1:** General characteristics of indigenous subtribes involved in the present study (N = 255).

Variables		Total (N = 255)		Temuan (N = 162)		Temiar (n = 93)	
		No examined	Percentage (%)	No examined	Percentage (%)	No examined	Percentage (%)
Age group	≤ 12	138	54.1	82	50.6	56	60.2
	> 12	117	45.9	80	49.4	37	39.8
Gender	Male	132	51.8	85	52.5	47	50.5
	Female	123	48.2	77	47.5	46	49.5
Socioeconomic Status							
Education	Formal	157	61.6	97	59.9	60	64.5
Household income	<RM 500	153	60.0	93	57.4	60	64.5
Family Size	<6	131	51.4	80	49.4	51	54.8
Working	Unemployed	186	72.9	112	69.1	74	79.6
Water source	Untreated water	156	61.2	121	74.7	35	37.6
Boil water	Yes	222	87.1	130	80.2	92	98.9
Presence of latrine	Yes	201	78.8	113	69.8	88	94.6
Defecation	Indiscriminate	167	65.5	84	51.9	83	89.2
Take bath in river	Yes	137	53.7	59	36.4	78	83.9
Garbage disposal	Proper place	179	70.2	99	61.1	80	86.0
Own Pets	Yes	183	71.8	103	63.6	80	86.0
Wear shoes outside of house	Yes	206	80.8	113	69.8	93	100.0

### Temuan and Temiar subtribes: Overall STH infections

The prevalence of STH amongst Temuan was 53.2% (of 171, 95% CI: 45.7–60.7), which consisted of *T. trichiura* (n = 79, 46.2% of 171, 95% CI: 38.7–53.7), *Ascaris* spp. (n = 44, 25.7%, 19.2–32.3) and hookworm (n = 7, 4.1%, 1.1–7.1) (Table 2). Single infection (n = 54; 31.6%, 24.6–38.6) was found to be the most common, followed by multiple infections (n = 37, 21.6%, 15.4–27.8). Among the single infection, the most prevalent was *T. trichiura* infections (n = 42; 24.6%, 18.1–31.1). The combination of *T. trichiura* and *Ascaris* spp. (n = 31, 18.1%, 12.3–23.9) was predominant in the multiple infections.

**Table 2:** Parasitic infections among the indigenous subtribes (N = 269).

Soil-transmitted helminth infection	Total (N = 269)			Temuan (n = 171)			Temiar (n = 98)		
	n positive	% positive	95% CI	n positive	% positive	95% CI	n positive	% positive	95% CI
**Overall Soil transmitted helminth infection**	149	55.4	49.4–61.0	91	53.2	45.7–60.7	58	52.7	45.8–62.6
**Helminth infection**									
*T. trichiura*	114	42.4	36.1–48.3	79	46.2	38.7–53.7	35	35.7	26.2–45.2
*Ascaris* spp.	83	30.9	25.7–36.4	44	25.7	19.2–32.3	39	39.8	30.1–49.5
Hookworm	15	5.6	3.0–8.6	7	4.1	1.1–7.1	8	8.3	2.8–13.8
**Mix infections**	**50**	**18.6**	**13.8**–**23.0**	**17**	**9.9**	**5.4**–**14.4**	**33**	**33.7**	**24.3**–**43.1**
**Helminth with:**									
**Single protozoa infection**	**44**	**16.4**	**19.0**–**28.3**	**16**	**9.4**	**5.0**–**13.8**	**28**	**28.6**	**19.7**–**37.6**
*Entamoeba* spp.	35	13.0	8.9–17.1	11	6.4	2.7–10.1	24	24.5	16.0–33.0
*Giardia* sp.	5	1.9	0.4–3.3	2	1.2	−0.4–2.8	3	3.1	−0.3–6.5
*Cryptosporidium* spp.	3	1.1	0.4–3.0	3	1.8	−0.2–3.8	-	-	-
*Sacrocystis* spp.	1	0.4	0.0–1.1	-	-	-	1	1.0	−0.9–3.0
**Multiple protozoa infections**	**6**	**2.2**	**1.1**–**5.2**	**1**	**0.6**	**−0.6**–**1.8**	**5**	**5.1**	**0.7**–**9.5**
*Entamoeba* spp.+*Giardia* sp.	5	1.9	0.4–3.7	-	-	-	5	5.1	0.7–9.5
*Entamoeba* spp.+*Cryptosporidium* spp.	1	0.4	0.0–1.1	1	0.6	−0.6–1.8	-	-	-

Prevalence of STH amongst the Temiar subtribe was 52.7% (of 98, 95% CI: 45.8–62.6), which is slightly lower than the Temuan. Based on 98 samples, most infections were caused by *Ascaris* spp. (n = 39, 39.8%, 95% CI: 30.1–49.5) instead of *T. trichiura* (n = 35, 35.7% of 98, 26.2–45.2), followed by hookworm (n = 8, 8.2%, 2.8–13.8) (Table 2). Single infection (n = 37, 37.8%, 28.2–47.4) was found to be the most common, followed by multiple infections (n = 21, 21.5%, 13.4–29.6). In contrast with the Temuan subtribe, *Ascaris* spp. was most prevalent in the Temiar subtribe (n = 19; 19.4%) for single infections. Similar to the Temuan, *T. trichiura* and *Ascaris* spp. (n = 13, 13.3%, 6.6–20.0) infections were still the predominant combination in multiple infections in the Temiar subtribe.

### Temuan and Temiar subtribes: STH and their co-infection with protozoa

In the Temuan subtribe, 17 (9.9% of 171, 95% CI: 5.4–14.4) were infected with both STH and protozoa. Evaluation of stool samples indicated double infections of STH with *Entamoeba histolytica/dispar/moshkovskii* (n = 11; 6.4%, 5.0–13.8), *Cryptosporidium* spp. (n = 3, 1.8%, −0.2–3.8) and *Giardia* spp. (n = 2, 1.2%, −0.4–2.8). One sample had co-infections of STH with *Entamoeba histolytica/dispar/moshkovskii* and *Cryptosporidium* spp. (n = 1; 0.6%, −0.6–1.8).

Co-infections of STH with other intestinal protozoa were also observed among the Temiar subtribe (n = 33; 33.7%, 95% CI: 24.3–43.1). Overall, the prevalence of co-infections of STH with protozoa was higher among the Temiar compared to Temuan. The most common co-infections was with *Entamoeba histolytica/dispar/moshkovskii* (n = 24; 24.5%, 16.0–33.0), followed by *Giardia* spp. (n = 3; 3.1%, −0.3–6.5). In comparison with Temuan, there was no *Cryptosporidium* spp. detected among the Temiar. However, it was interesting to note that there was a case of intestinal *Sarcocystic* spp. (n = 1; 1.0%, −0.9–3.0) detected in a Temiar individual. The affected individual did not seem to present with any symptoms such as muscle ache and tissue biopsy was not performed on this individual to rule out muscular sarcocystosis. There were five samples which had co-infections of STH with *Entamoeba histolytica/dispar/moshkovskii* and *Giardia* spp. (n = 5; 5.1%, 0.7–9.5).

### Temuan and Temiar subtribes: STH and their association with risk factors

For the Temuan subtribe, four variables were found to be significantly associated with STH infections and these include age group less than or equal to 12 years old (OR = 2.23; 95% CI = 1.19–4.19), household income less than RM500 (OR = 2.54; 95% CI = 1.34–4.82), being unemployed (OR = 2.23; 95% CI = 1.13–4.41) and indiscriminate garbage disposal (OR = 2.40; 95% CI = 1.25–4.63) (Table 3). Using multivariate analysis, it was also further confirmed that age group less than or equal to 12 years old (OR = 2.04; 95% CI = 1.06–3.92), household income less than RM500 (OR = 2.09; 95% CI = 1.07–4.08) and indiscriminate garbage disposal (OR = 2.01; 95% CI = 1.01–4.00) were the risk factors for STH infections among the Temuan (Table 4).

**Table 3:** Associations between soil-transmitted helminthiasis and risk factors among the indigenous subtribes(N = 255).

Variables		Total (N = 255)				Temuan (N = 162)				Temiar (n = 93)			
		No examined	Infected (%)	OR (95% CI)	p value	No examined	Infected (%)	OR (95% CI)	p value	No examined	Infected (%)	OR (95% CI)	p value
**Age groups**	≤ 12	138	65.9	2.51 (1.51−4.16)	0.000	82	63.4	2.23 (1.19–4.19)	0.012	56	69.6	3.01 (1.27–7.15)	0.011
	> 12	117	43.6	1		80	43.8	1		37	43.2	1	
**Gender**	Male	132	54.5	1.10 (0.67–1.81)	0.704	85	52.9	1.07 (0.58–1.98)	0.838	47	57.4	1.15 (0.50–2.64)	0.737
	Female	123	56.9	1		77	54.5	1		46	60.9	1	
**Socioeconomic Status**													
Household income	<RM 500	153	60.8	1.68 (1.01–2.78)	0.045	93	63.4	2.54 (1.34–4.82)	0.004	60	56.7	0.75 (0.31–1.79)	0.513
	≥ RM 500	102	48	1		69	40.6	1		33	63.6	1	
Family Size	<6	131	52.7	1.30 (0.78–2.11)	0.319	80	52.5	1.10 (0.59–2.04)	0.762	51	52.9	1.78 (0.76–4.14)	0.18
	≥ 6	124	58.9	1		82	54.9	1		42	66.7	1	
Working	Unemployed	186	60.8	2.14 (1.22–3.74)	0.007	112	59.8	2.23 (1.13–4.41)	0.019	74	62.2	1.83 (0.66–5.04)	0.242
	Employed	69	42	1		50	40	1		19	47.4	1	
Water source	Untreated water	156	51.9	0.67 (0.40–1.12)	0.129	121	51.2	0.67 (0.33–1.38)	0.280	35	54.3	0.73 (0.31–1.70)	0.459
	Treated water	99	61.6	1		41	61	1		58	62.1	1	
Presence of latrine	No	54	57.4	1.09 (0.60–2.01)	0.774	49	57.1	1.22 (0.62–2.40)	0.563	5	60	1.04 (0.17–6.53)	0.968
	Yes	201	55.2	1		113	52.2	1		88	59.1	1	
Defecation	Indiscriminate	167	59.3	1.52 (0.91–2.56)	0.111	84	60.7	1.80 (0.97–3.37)	0.063	83	57.8	0.59 (0.14–2.43)	0.46
	Proper place	88	48.9	1		78	46.2	1		10	70	1	
Take bath in river	Yes	137	60.6	1.54 (0.93–2.53)	0.09	59	54.2	1.03 (0.54–1.97)	0.918	78	65.4	5.19 (1.51–17.88)	0.005
	No	118	50	1		103	53.4	1		15	26.7	1	
Garbage disposal	Indiscriminate	76	67.1	1.97 (1.13–3.46)	0.017	63	66.7	2.40 (1.25–4.63)	0.008	13	69.2	1.66 (0.47–5.85)	0.425
	Proper place	179	50.8	1		99	45.5	1		80	57.5	1	
Own Pets	Yes	183	54.1	0.80 (0.46–1.38)	0.416	103	48.5	0.56 (0.29–1.08)	0.082	80	61.3	1.84 (0.57–6.00)	0.304
	No	72	59.7	1		59	62.7	1		13	46.2	1	

**Table 4:** Multivariate analysis of risk factors associated with STH infections among the indigenous subtribes (N = 255).

Variables		OR (95% CI)	p- value
**a. Overall indigenous community**			
Age groups	≤ 12	2.51 (1.50–4.19)	0.000
Garbage disposal	Indiscriminate	1.98 (1.11–3.51)	0.020
**b. Temuan**			
Age groups	≤ 12	2.04 (1.06–3.92)	0.033
Household income	<RM500	2.09 (1.07–4.08)	0.030
Garbage disposal	Indiscriminate	2.01 (1.01–4.00)	0.046
**c. Temiar**			
Take bath in river	No	5.65 (1.55–20.57)	0.009

As for the Temiar, univariate analysis identified two factors that were significantly associated with STH infections. These factors include age less than or equal to 12 years old (OR = 3.01; 95% CI = 1.27–7.15), and the practice of taking bath in the river (OR = 5.19; 95% CI = 1.51–17.88) (Table 3). Furthermore, multivariate analysis confirmed that the practice of taking bath in the river (OR = 5.65; 95% CI = 1.55–20.57) was a risk factor for STH infections (Table 4).

## Discussion

The current results demonstrated that 53.2% of Temuan were infected with at least one STH, whereas 52.7% in the Temiar subtribe. This was in concordance with a study by Dunn [13] whereby it was reported that Orang Asli that live outside or close to the town acquired higher rates of intestinal parasitic infections compared to Orang Asli that are still depend on forest dwelling or traditional agriculturists. In the present study, the Temiar subtribe lives at the inner forest compared to the Temuan subtribe who live closer to town and cities.

Most of the recent studies have demonstrated that *T. trichiura* was the most prevalent (range: 26.0% to 98.2%) parasite in Malaysia especially among the Orang Asli communities in recent years [2], [6], [15], [22]–[28]. Similarly, the current finding highlighted that the infection of *T. trichiura* to be the highest among Temuan (46.2% of 171). Some reasons that have been pinpointed by other studies in Malaysia included that *T. trichiura* possesses higher rate of infections due to its resistance to anthelminthics, ineffective dosage and choice of anthelminthic used [1], [2], [29], [30]. Local studies showed that a single dose (400 mg) of albendazole was proven ineffective to kill *T. trichiura* [29]–[31]. Instead, increased doses of albendazole were required in order to obtain higher cure rate among the infected persons [1], [31].

In contrast, *Ascaris* spp. was the most common STH among Temiar subtribe (39.8% of 98) instead of *T. trichiura*. Based on single STH infection, *Ascaris* spp. was three times higher in the Temiar compared to Temuan. This result was in agreement to available information found in the late 1990s, which indicated that *Ascaris* spp. infections were the most common STH (47.5% to 59.5%) [32]–[34]. Since Temiar subtribe live further away from urban/developed areas, they may have lesser opportunity to partake in deworming programmes compared to the Temuan subtribe. Contrary to *T. trichiura*, studies proved that single oral dose of albendazole (400 mg) showed 88.0%–100% cure rate against *Ascaris* spp. [29], [30], [35]–[37]. Mass drug administration should be applied periodically to these communities who are living in rural areas in order to curtail *Ascaris* spp. and also other STH transmission.

As shown in the results of the current study, mix infections of STH and intestinal protozoa parasites was almost four times higher among the Temiar (33.7% of 98) compared to the Temuan (9.9% of 171). *Entamoeba histolytica/dispar/moshkovskii* was the most predominant protozoa among the two subtribes and the prevalence was 29.6% in Temiar and 7.0% in Temuan subtribes. Our result was in agreement with the most recent study carried out among the three ethnic groups of Orang Asli and the prevalence of *Entamoeba histolytica/dispar/moshkovskii* was reported as 29.5% among the Jahai subtribe (under the Negrito category), 18.5% among the Jahut subtribe (Senoi) and 8.7% among the Temuan subtribe (Proto-Malay or Aboriginal Malay) [20]. Their results reported that Senoi had higher prevalence of *Entamoeba histolytica/dispar/moshkovskii* compared to the prevalence in Proto-Malay, main ethnicity is Temuan from Parit Gong village, Jelebu, Negeri Sembilan. As discussed in Anuar et al. [20], this trend can be due to better housing condition and provision of basic amenities among Proto-Malay compared to Senoi. As *Entamoeba histolytica* can cause amoebiasis which leads to human mortality [38], public health authorities should implement appropriate preventive and control plans to reduce the *Entamoeba histolytica/dispar/moshkovskii* infections among these indigenous communities.

In addition, a rarely detected protozoa, *Sarcocystis* spp. was also isolated in this study. This parasite was found in one of the Temiar individual (1.0% of 98), an asymptomatic 20 year old housewife in Pos Piah, Perak. The infected female was staying at the inner forest, around 60 km from town. To the best of our knowledge, this is one of the very few reports of intestinal *Sarcocystis* spp. (human is the definitive host) found in human in Malaysia. This protozoan parasite normally undergoes heteroxenous intermediate-definitive host life cycle [39]. Thusfar, there have been only two studies of *Sarcocystis* spp. reported in the Orang Asli [16], [17]. The first incidental findings of sarcocystosis was found in the muscle of the oropharyngeal region in an indigenous girl in 1975 [16], followed by the second publication in 1978 which was diagnosed using a fluorescent antibody technique [17]. The latter study indicated that the highest prevalence of *Sarcocystis* sp. was found among the indigenous community (39.9%), followed by Malays (17.0%), Indians (8.7%) and Chinese (3.6%) [17]. Although report on sarcocystosis is scanty among Malaysians, recently in 2011–2012, outbreaks of acute muscular *Sarcocystis*-like infection were reported among international travellers returning from Tioman Island, Malaysia [40], [41]. Another outbreak in 2012 happened among college students associated with myalgia and myositis in another Malaysian island, i.e., Pangkor island [42].

Compared to Temuan, most of Temiar have to engage on self-sufficient farming to support their livelihood due to low household income. As for farming, they set up traps around the farm in order to prevent wild animals from destroying their farm. At the same time, animals that are caught in these cages usually end up as their food, as they have limited means to go to town to buy meat. In Malaysia, *Sarcocystis* cyst have been reported from various nonhuman primates such as rodent [43]–[48], slow loris [49], buffalo [50]–[52], long-tailed macaque [53], [54], snake [55], cattle [52] and zoo animals [56]. With the exception of the study conducted by Lau et al. [55], all human and non-human studies in Malaysia were regarded as muscular sarcocystosis. Therefore, we postulated that the infected person in the present study might have consumed the sarcocysts in uncooked or raw meat from these captured infected animals in the farm (e.g. rats, monkey or wild boars), which also have proven to act as intermediate host for *Sarcocystis* spp. [47], [53], [57]. Since these animals were allowed to roam freely and defecate indiscriminately around the compound, they could have acquired the oocysts or free sporocysts through the contaminated environment. Usually, most Temiar cook their food thoroughly before consuming it. Unfortunately, the limitation of the study was that we were unable to confirm this postulation due to logistic and financial constraints.

Although this may be the first study reporting on intestinal sarcocystosis in human, it is predicted that this parasite may be endemic in this country. *Sarcocystis* spp. that was found in most of the previous studies was incidental findings. Therefore, it is predicted that the prevalence of this parasite in Malaysia is underestimate due to the lack of studies focusing on *Sarcocystis* spp. Future studies should incorporate molecular characterization of *Sarcocystis* spp. in order to confirm the species and route of transmission. Currently, the epidemiology of human *Sarcocystis* spp. is still poorly investigated. *Sarcocystis* spp. outbreaks occurred for two years continuously in Malaysia [41] and these outbreaks have brought international attention to the importance of this parasite. This protozoan parasite can become a severe public health problem in future if the sources of infection are not monitored or controlled strictly [58]. Although there is only one case of *Sarcocystis* infection found in the current study, heighten awareness and more research activities should be initiated to understand this parasite better. Preventive measures such as boil water, wash or cook food thoroughly should also be practiced among indigenous communities in order to prevent humans, who may be definitive host of *Sarcocystis* from infecting and transmitting this infection to others.

As conclusion, the present study highlighted the importance of understanding the variation of infections amongst the different Orang Asli subtribes. It is vital to note these differences and use this knowledge to customise effective control measures for the various subtribes. In addition, promoting health education based on the need of the specific subtribe is another powerful measure that can heighten health awareness and thus produce significant behavioural changes in the effort to reduce mortality of parasitic infections. Furthermore, molecular genotyping of helminthic infections especially *T. trichiura* and *Ascaris* spp. should be included in future study to obtain better understanding of the differences between subgroups and also with other races in Malaysia.
